# Current Perspectives on Pyrroloiminoquinones: Distribution, Biosynthesis and Drug Discovery Potential

**DOI:** 10.3390/molecules27248724

**Published:** 2022-12-09

**Authors:** Jarmo-Charles J. Kalinski, Alexandros Polyzois, Samantha C. Waterworth, Xavier Siwe Noundou, Rosemary A. Dorrington

**Affiliations:** 1 Department of Biochemistry and Microbiology, Rhodes University, Makhanda 6140, South Africa; 2 Division of Pharmaceutical Sciences, University of Wisconsin, Madison, WI 53705, USA; 3 Department of Pharmaceutical Sciences, School of Pharmacy, Sefako Makgatho Health Sciences University, Pretoria 0208, South Africa; 4 South African Institute for Aquatic Biodiversity, Makhanda 6140, South Africa

**Keywords:** makaluvamine, damirone, discorhabdin, batzelline, tsitsikammamine, epinardin, prianosin, Latrunculiidae, Acarnidae, sponges

## Abstract

Pyrroloiminoquinones are a group of cytotoxic alkaloids most commonly isolated from marine sponges. Structurally, they are based on a tricyclic pyrrolo[4,3,2-de]quinoline core and encompass marine natural products such as makaluvamines, tsitsikammamines and discorhabdins. These diverse compounds are known to exhibit a broad spectrum of biological activities including anticancer, antiplasmodial, antimicrobial, antifungal and antiviral activities as well as the inhibition of several key cellular enzymes. The resurgence of interest in pyrroloiminoquinones and the convoluted understanding regarding their biological activities have prompted this review. Herein, we provided a concise summary of key findings and recent developments pertaining to their structural diversity, distribution, biogenesis, and their potential as chemical probes for drug development, including a discussion of promising synthetic analogs.

## 1. Introduction

Pyrroloiminoquinones are a large and diverse group of natural products that have been isolated predominantly from marine sponges [1,2,3,4]. They are considered to be potential drug leads due to their significant inhibition of cell proliferation in various cancer cell lines, including promising in vivo activity against several tumor types [5,6,7,8,9] and inhibition of *Plasmodium berghei* parasitemia [10] in mouse models. In addition, pyrroloiminoquinones have been shown to exhibit antiviral [11,12], antifungal [13,14,15] and antibacterial [5,12,15,16,17] as well as neuromodulatory [18,19] and antioxidant [20] activities. The mechanisms of bioactivity for these compounds are not yet completely understood with members of this compound class appearing to exert their activity through a number of different modes of action which, for anticancer cell activity, include direct DNA damage [7,8] and inhibition of key cell regulatory enzymes [21].

**Figure 1:**
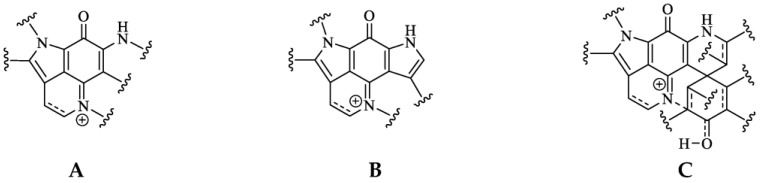
Pyrroloiminoquinone structural scaffolds (**A**)—Makaluvamines, (**B**)—Bispyrroloiminoquinones, (**C**)—Discorhabdins.

Pyrroloiminoquinone molecular structures are characterized by a condensed tricyclic pyrrolo[4,3,2-de]quinoline core that is also considered the principal pharmacophore of this compound class responsible for their antiproliferative and cytotoxic effects [7,22]. Most compounds of this class can be assigned to one of three major classes exhibiting distinct core structures, namely makaluvamines, bispyrroloiminoquinones and discorhabdins (Figure 1).

Pyrroloiminoquinones have been mostly isolated from marine sponges of the order Poecilosclerida, with Latrunculiidae species from temperate and cold-water environments such as New Zealand, South Africa, the Arctic, and Antarctic as well as warm-water Acarnidae species from the Indo-Pacific proving particularly productive sources [2,3,4]. Nevertheless, members of this class of alkaloids have been reported from ascidians [23,24] and simple representatives have also been isolated from cultured myxomycetes [25,26]. Moreover, closely related alkaloids have been reported in hydroids [27,28], terrestrial fungi [29,30,31,32] and marine actinobacteria [33,34]. This wide geographical and phylogenetic distribution of pyrroloiminoquinone producers, as well as the production of related compounds by bacteria raises the question of microbial involvement in their biosynthesis within marine invertebrates.

Inspired by the natural products, several types of pyrroloiminoquinone analogs have been synthesized over the years, leading to potential drug leads, some of which showed promising in vivo anticancer profiles. Synthetic protocols have been developed for many pyrroloiminoquinones and related analogs. A detailed discussion of their chemical synthesis is beyond the scope of this review and we refer the interested reader to several focused articles [35,36,37]. This review aimed to summarize the chemical diversity of pyrroloiminoquinones, and related compounds isolated to date, as well as the current state of knowledge pertaining to their biosynthetic origin and the potential these remarkable alkaloids hold as potential drug leads.

## 2. Structures and Host Distribution of Natural Pyrroloiminoquinones and Related Compounds

### 2.1. Makaluvamines, Bispyrroloiminoquinones and Discorhabdins

**Figure 2:**
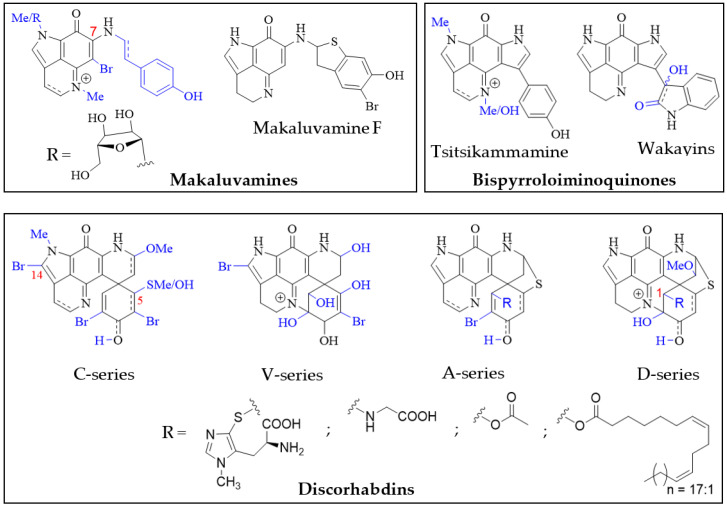
Main pyrroloiminoquinone classes. Variable substituents encompassing all known compound class members are shown in blue.

Structurally, the simplest pyrroloiminoquinones are represented by the makaluvamines consisting of the characteristic pyrrolo[4,3,2-de]quinoline core and variable substituents (Figure 2). These include *N*-methylation of the pyrrole or imine nitrogen, halogenation at C-6, Δ3,4-desaturation and alkylation of N-7 with phenylethyl based side chains. Notable exceptions are makaluvamine O and makaluvamine W and the broad use of the term ‘makaluvamine’ in this review excludes these two structures. Makaluvamines were first reported in 1993 in the sponge *Zyzzya fuliginosa* collected near the Makaluva Islands, Fiji [7] and since then, have most routinely been isolated from Pacific and Indo-Pacific warm-water sponges of the genus *Zyzzya* (family Acarnidae) [8,10,11,20,38,39,40,41,42,43,44,45,46,47]. Makaluvamines have also been isolated from latrunculid sponge species collected off South Africa [22,48,49], the Korean peninsula [16], New Zealand [50] and Australia [21]. Interestingly, simple makaluvamines bearing either no substituents or only exhibiting *N*-methylation have been purified from cultured myxomycetes, *Didymium iridis* and *Didymium bahiense*, isolated from Japanese forest litter samples [25,26]. Makaluvamines are thought to be the biosynthetic precursors to more complex pyrroloiminoquinones and the sulfur-containing makaluvamine F may represent a precursor to sulfur-containing discorhabdins [50].

Bispyrroloiminoquinones are relatively rare pyrroloiminoquinones containing a characteristic pyrrolo[4,3,2-de]pyrrolo[2,3-h]quinoline core and, depending on the nature of the side-chain, they can be classed as either tsitsikammamines or wakayins (Figure 2). Tsitsikammamines have been reported in the South African marine sponges *Tsitsikamma favus* and *Tsitsikamma nguni* [15,22,49,51,52], an Australian *Zyzzya* sp. [45], Tongan *Strongylodesma tongaensis* [53] as well as Antarctic *Latrunculia biformis* [54]. Wakayins (wakayin and 16-hydroxy-17-oxyindolewakayin) on the other hand, have only ever been found in ascidians of the genus *Clavelina* collected in Micronesia (Wakaya Islands) [23] and Thailand [24].

Pyrroloiminoquinones comprising a pyrido[2,3-h]pyrrolo[4,3,2-de]quinoline core with an additional spiro-fused cyclohexanone/-ol or cyclohexadienone/-ol moiety are known as discorhabdins (Figure 2), some of which are historically also referred to as epinardins or prianosins [55,56,57]. Members of this structurally complex and diverse class of pyrroloiminoquinones are known for particularly potent bioactivities and have attracted significant interest from natural product, medicinal and synthetic chemists alike [5,6,7,9,12,14,15,16,18,21,22,35,36,37,54,58,59,60,61,62,63,64,65,66,67,68,69,70,71,72,73,74,75]. In contrast to makaluvamines and bispyrroloiminoquinones, discorhabdins have been exclusively found in demosponges, mostly belonging to the family Latrunculiidae. While they all share a characteristic spiro-arrangement flanking the pyrroloiminoquinone core, most known discorhabdins can be divided into distinct structural sub-classes: First, the often multi-brominated, pentacyclic discorhabdins of the C-series and analogous hexacyclic discorhabdins of the V-series exhibiting N(18)-C(2) ring closure and, second, the hexacyclic discorhabdins of the A-series with a bridging sulfur atom connecting C-5 and C-8 and analogous heptacyclic discorhabdins of the D-series displaying N(18)-C(2) ring closure. Some sponges, such as those of genus *Tsitsikamma* [15,22,49,52], produce only C- and V-series discorhabdins, while the presence of A- and D-series discorhabdins is often accompanied by discorhabdins of the former classes. C-14 bromination also appears to be particular to *Tsitsikamma* sponges [15,22,49,52] and may represent a chemotaxonomic distinction, while C-5 thiomethylation has only been observed in isolates from a single Caribbean deep-water *Strongylodesma purpureus* sponge [76] (reassigned from *Batzella* [77]). A- and D-series discorhabdins often exhibit substitution at C-1 with substituents such as ovothiol, glycine and alkyl esters [22,66,68,72], possibly as a result of increased electrophilic reactivity of the parent compounds [70]. Furthermore, A- and D-series discorhabdins are chiral and enantiomers of opposite parity have been isolated from the same *Latrunculia* species collected in different locations [65]. To date, all evidence available for comparison suggests that enantiomeric parity does not significantly affect biological activity [65,72].

### 2.2. Unusual Pyrroloiminoquinones and Related Pyrroloquinolines from Marine Sponges

**Figure 3:**
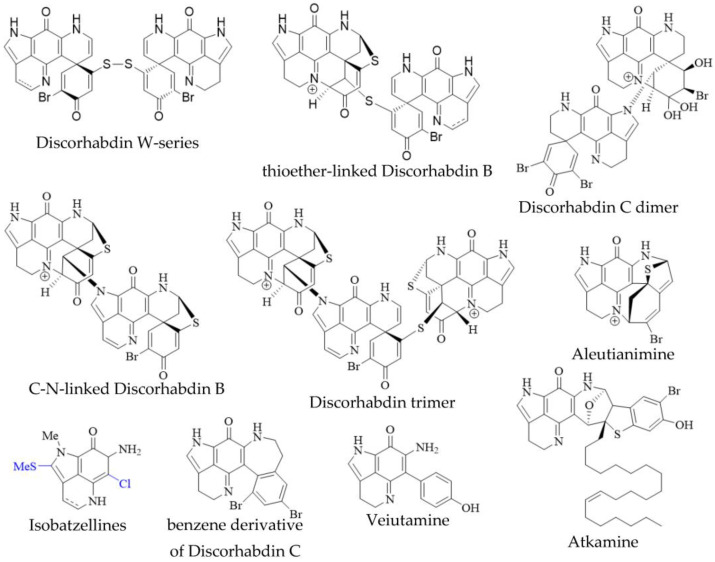
Unusual pyrroloiminoquinones from marine sponges. Variable substituents are shown in blue.

In addition to the monomeric discorhabdins discussed above, several dimeric and trimeric discorhabdins have been isolated from sponges of the genus *Latrunculia* (Figure 3). These comprise W-series discorhabdins characterized by a disulfide bridge linking two discorhabdin monomers [64,66], thioether-linked discorhabdin dimers [70,74], a C-N-linked discorhabdin C dimer [73], a C-N-linked discorhabdin B dimer as well as a discorhabdin trimer [75]. The saturated discorhabdin W dimer and its monomers have been shown to be interconvertible through reductive cleavage and subsequent UV-irradiation [64], whereas thioether-linked discorhabdin dimers were first discovered as a major degradation product of monomeric discorhabdin stored at −20 °C for a fortnight [70]. Such non-enzymatic dimerization together with the observations that discorhabdins have been shown to be prone to nucleophilic attack at C-1 [70,73] suggest that at least sulfur-bridged discorhabdin dimers may be generated non-enzymatically in situ [73]. Furthermore, LC-MS/MS-driven molecular networking has provided evidence for numerous discorhabdin di- and trimers, some even incorporating makaluvamines, alongside monomeric A- and D-series discorhabdins in extracts of subantarctic *Latrunculia apicalis* and South African *Cyclacanthia bellae* [52].

Alike to oligomeric discorhabdins exemplifying a special case of discorhabdin structures, isobatzellines are makaluvamine-like structures that are distinguished by thiomethylation at C-2 or chlorination at C-6 and can thus be regarded as a subgroup of makaluvamines (Figure 3). Isobatzellines exhibiting thiomethylation have been exclusively reported in a Grand Bahaman *Strongylodesma nigra* specimen [13] (reassigned from *Batzella* [77]), while those only containing chlorine substituents have also been purified from extracts of Australian and Indopacific *Zyzzya* sponges [8,11]. Furthermore, two exotic pyrroloiminoquinones, atkamine and aleutianimine (Figure 3), have been isolated from Alaskan *Latrunculia* sp. [78] and *Latrunculia austini* [79], respectively. The structure and stereochemistry of atkamine were secured through chemical degradation, as well as comparison of experimental and TDDFT-simulated ECD spectra. Using a similar toolset incorporating DFT-simulated NMR spectra, the same group verified the structure of aleutianimine, providing a compelling example of the usefulness of computational approaches in natural product structure elucidation. Other unusual pyrroloiminoquinones are represented by the benzene derivative of discorhabdin C, isolated alongside various discorhabdins from an Alaskan deep-water *Latrunculia* sp. [12]. The same compound has been afforded semi-synthetically through dienol-benzene rearrangement [14] and is therefore considered to likely be an isolation artifact [12]. Veiutamine was isolated as a minor secondary metabolite alongside several common pyrroloiminoquinones from Fijian *Z. fuliginosa* and showed potent in vitro cytotoxicity in a panel of 25 cancer cell lines [80]. Veiutamine exhibits an unusual phenol-substituent directly bound to the pyrroloiminoquinone core and is to date the only known pyrroloiminoquinone with a C-6 *p*-oxy benzyl substituent.

**Figure 4:**
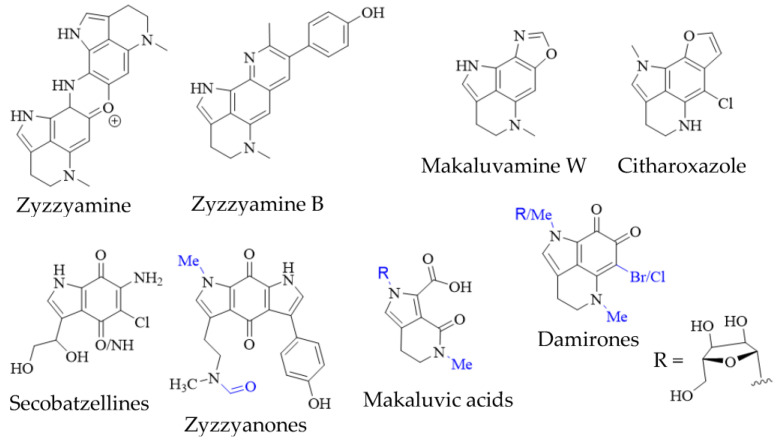
Pyrroloiminoquinone-related marine natural products from sponges. Variable substituents are shown in blue.

In addition to true pyrroloiminoquinones, several closely related natural products have been isolated from marine sponges, often as minor secondary metabolites alongside makaluvamines, discorhabdins and tsitsikammamines (Figure 4). They include the secobatzellines from a Caribbean *Strongylodesma* sp. sponge [19] (reassigned from *Batzella* [81]); zyzzyanones from Australian *Z. fuliginosa* [45,82]; makaluvic acids from Micronesian *Z. fuliginosa* [40] and South African *Strongylodesma aliwaliensis* [83]; the oxazole-containing makaluvamine W from Tongan *Strongylodesma tongaensis* [53]; the structurally related citharoxazole from Mediterranean *Latrunculia citharistae* [84] and zyzzyamines from Papua New Guinean *Z. fuliginosa* [85].

Pyrroloiminoquinone isolations commonly result in the recovery of *ortho*-quinone analogs of makaluvamines, named damirones [86] (Figure 4; some analogs are referred to as batzellines [87] and this structural archetype also encompasses makaluvamine O). As a result, such pyrrolo-*ortho*-quinones have been isolated from various makaluvamine- and isobatzelline-producing marine sponges including *C. bellae* (previously *Latrunculia bellae* [22,88]), *S. aliwaliensis* [48], *Spongosorites* sp. [67], *Smenospongia aurea* [89,90], *Zyzzya* spp. [7,8,39,41,42,45,46] and *T. favus* [49], in addition to the myxomycete *D*. *iridis* [26]. These compounds generally show greatly decreased cytotoxicity compared to e.g., makaluvamines that contain a pyrroloiminoquinone core [7,22]. Their formation from makaluvamines has been shown to be possible through, alkaline hydrolysis, lyophilization [91] and UV irradiation [92]. This, together with the fact that they in most cases have been isolated alongside makaluvamines, suggests that they may arise simply as degradation products. However, it can currently not be excluded that they may occupy a functional role in pyrroloiminoquinone biosynthesis.

### 2.3. Pyrroloiminoquinones and Related Pyrroloquinolines from Hydroids, Bacteria and Fungi

**Figure 5:**
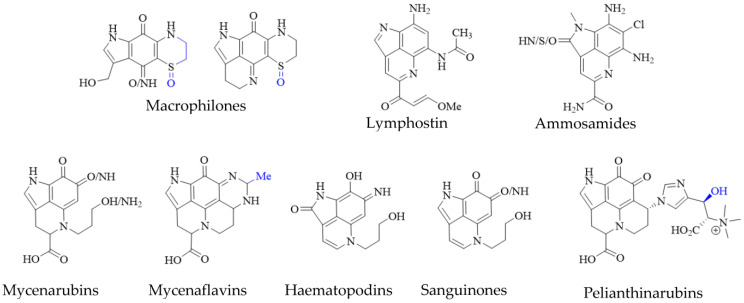
Pyrroloiminoquinone-related natural products from marine hydroids, marine soil-derived bacteria, and terrestrial fungi. Variable substituents are shown in blue.

Pyrroloiminoquinone-related compounds have been isolated from several organisms unrelated to marine sponges or ascidians and this occurrence may hold important information to aid the identification of biosynthetic gene clusters or possibly even microbial symbionts responsible for or involved in pyrroloiminoquinone biosynthesis. Such organisms include the Australian marine hydroid *Macrorynchia philippina* which contains several cytotoxic macrophilones [27,28]. Some of these, exhibit a fully formed pyrroloiminoquinone core and have been shown to inhibit the conjugation of SUMO peptides to target proteins, eliciting greatly decreased levels of proteins involved in ERK signaling, while also exhibiting selective cytotoxicity in the NCI-60 anticancer panel [27,28]. In addition, related pyrroloquinoline alkaloids such as the lymphocyte kinase-inhibiting lymphostin [33] and the selectively cytotoxic ammosamides [34] are produced by marine-derived actinomycetes, *Salinispora* sp. and *Streptomyces* sp., respectively (Figure 5).

While the vast majority of pyrroloiminoquinone structures have been reported from marine sources, terrestrial fungi of the genus *Mycena* have been shown to produce a range of pigments with clear structural relation to pyrroloiminoquinones (Figure 5). These comprise mycenarubins, mycenaflavins, haematopodins [30,32], sanguinones [29] and pelianthinarubins [31]. Some mycenaflavins have shown cytotoxic activity, while haematopodins and mycenarubins have been reported to exert antibiotic activity against soil bacteria [32]; however, compared with marine pyrroloiminoquinones, the biological activity of these fungal pyrroloquinolines has not been extensively investigated to date.

## 3. Biosynthesis

**Figure 6:**
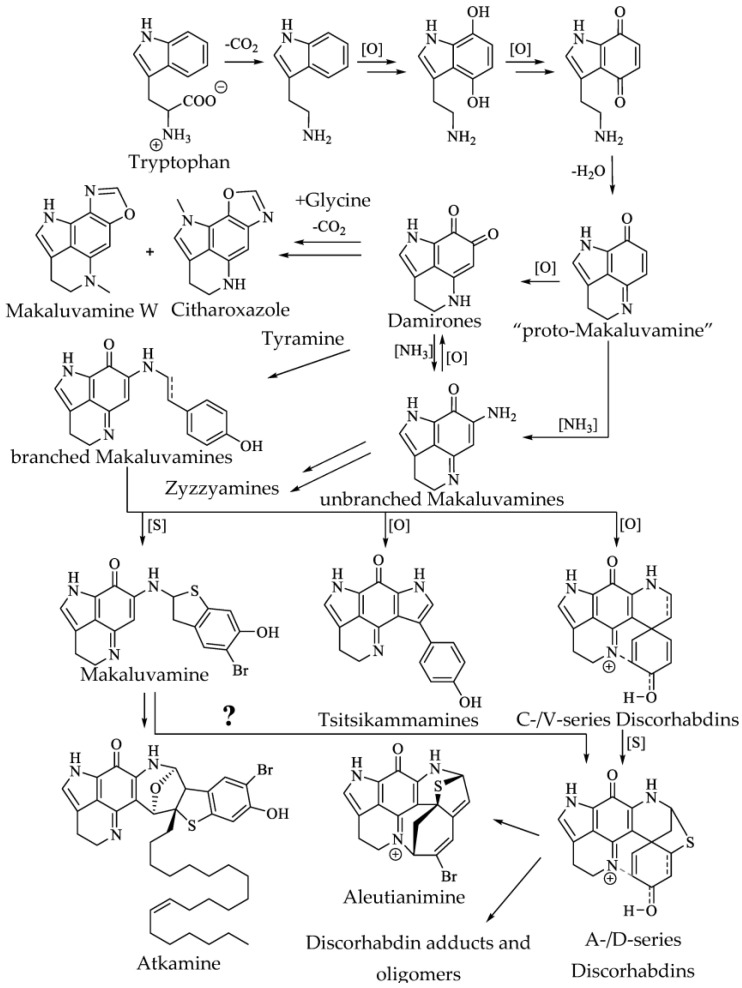
Putative biosynthetic pathway of pyrroloiminoquinones. Adapted from references [1,2,3,4,53,84,85].

The biosynthetic pathway of pyrroloiminoquinones remains largely hypothetical, yet chemotaxonomic relationships, structural similarities among the pyrroloiminoquinones and some direct experimental evidence have led to the proposal of a rational sequence of biosynthetic reactions [1,2,50] (Figure 6). Biosynthesis is proposed to begin with decarboxylation of tryptophan, followed by several oxidation steps and condensation to give a “proto”-makaluvamine. From this precursor, the proposed pathway proceeds either by oxidation or amination to yield pyrrolo-*ortho*-quinones or unbranched makaluvamines [50]. It is not clear whether the ortho-quinones are simply decomposition products of pyrroloiminoquinones or are produced biosynthetically, potentially as precursors or byproducts in makaluvamine biosynthesis.

The rare oxazole pyrroloiminoquinones makaluvamine W and citharoxazole have been proposed to derive from damirones by condensation with glycine, followed by decarboxylation and oxidation [53,84]. Zyzzyamines have been suggested to originate from similar condensation reactions of makaluvamines with alternative reaction partners [85].

The conversion of unbranched to branched makaluvamines is considered to incorporate tyramine. The sequence of tyramine addition and cyclization to more complex pyrroloiminoquinones is still unknown; however, discorhabdin B biosynthesis in tissue slices of a *Latrunculia* sp. sponge was shown to incorporate radio-actively labelled {U-14C}-L-phenylalanine [50], phenylalanine being a direct tyramine precursor. The mechanism by which *N*-methyl groups and halogens are incorporated into makaluvamines and discorhabdins is yet to be elucidated and the relevant reactions may not be confined to a specific point along the pyrroloiminoquinone biosynthetic pathway.

More complex pyrroloiminoquinones such as discorhabdins and tsitsikammamines are likely derived from makaluvamines bearing the *N*-phenylethyl side chain [50]. In support of this hypothesis, a possibly biomimetic synthetic sequence from makaluvamines to discorhabdins was reported in 1999 [93], in which tyramine derivatives were linked to a synthetic C-7 methoxy substituted pyrroloiminoquinone core to yield *N*-phenylethyl substituted makaluvamines followed by intramolecular cyclization to the corresponding discorhabdins. Discorhabdins bearing the C(5)–C(8) sulfur bridge, such as those of the A- and D-series, have been proposed to be biosynthesized from either makaluvamine F or from C-series discorhabdins [1,2,50]. Even less is understood about the biosynthesis of atkamine and aleutianimine, however, atkamine has been suggested to be derived from a *N*-phenylethyl makaluvamine, possibly even makaluvamine F [78], whereas the highly strained aleutianimine has been proposed to be a downstream product of discorhabdin A [79].

The biosynthetic origins of pyrroloiminoquinones remain a point of contention and debate. The study conducted by Lill et al. [50], where slices of *Latrunculia* sp. sponge were soaked in broad-spectrum antibiotics, suggested that discorhabdins were made by the sponge itself, rather than associated microbes. However, the broad distribution of pyrroloiminoquinones across various sponges, ascidians, and terrestrial myxomycetes has led the hypothesis that the makaluvamine core may be microbially produced and the host organism is responsible for the conversion to more complex pyrroloiminoquinones, such as discorhabdins and tsitsikammamines [49,94]. The conservation of abundant populations of Tethybacterales and Spirochetes in various pyrroloiminoquinone-producing latrunculid sponges collected from the South African coast and Bouvet Island in the Southern Ocean, highlights them as potential producers of makaluvamine precursors [49,94,95,96]. Since relatives of the Tethybacterales are found in abundance in several other taxonomically unrelated sponge species that do not produce makaluvamines, it appears more likely that the conserved Spirochetes may be involved in the production of makaluvamines. Previous studies into structurally related pyrroloquinoline alkaloids, lymphostins and ammosamides, identified a RiPP class gene cluster as the biosynthetic origin of these compounds [97]. Recently, it has been shown that ammosamides are biosynthesized by attachment of tryptophan to the C-terminus of a ribosomally synthesized peptide, followed by hydroxylations and oxidation to quinones, while the primary amine is then introduced from a glycine residue [98]. The pyrroloiminoquinone core could be biosynthesized in a similar fashion but this remains yet to be elucidated.

## 4. Drug Discovery Potential of Pyrroloiminoquinones

Many studies have confirmed the cytotoxicity of makaluvamines and discorhabdins against cancer cell lines, and in several cases the inhibition of important enzymes and expression of genes involved in regulating cell proliferation and stress responses have been implicated in their activity [9,19,21]. Preclinical in vivo tumor xenograft studies in mice have been carried out for a select few natural makaluvamines and discorhabdins [5,6,7,8,9], as well as two synthetic makaluvamine analogs [99,100,101], indicating potential applications against skin, pancreatic, prostate, breast and ovarian cancer as well as leukemia. In addition, several pyrroloiminoquinones have been identified as antimalarial drug leads, some showing promising in vivo activity in mice models [10]. Antimicrobial [16,62], antiviral [11] and antioxidant [20] activities have also been reported for this compound class, indicating a broad application potential for these remarkable alkaloids. This notion is reinforced by the presence of a patent from the 1990s and a recent patent application both claiming the application of various natural makaluvamines and related semi-synthetic derivatives as antineoplastic and antibacterial agents [17,102]. At times, unselective cytotoxicity has diminished the enthusiasm for pyrroloiminoquinones as potential drug leads, however several members of this compound class have shown selective activity which appears to be related to a decreased electrophilic reactivity for some of the respective compounds.

### 4.1. Cytotoxicity and Anticancer Potential

Pyrroloiminoquinones have been attracting considerable interest as potential anticancer drug leads; however, they do not appear to act uniformly on one particular target, but rather, several mechanisms are responsible for their bioactivity depending on their particular structure. These include topoisomerase I and II inhibition [7,8,22,49], induction of apoptosis [99,100,101] and inhibition of key stress regulatory enzymes such as HIF-1α [21]. The principal pharmacophore of this compound class is represented by the pyrrolo[4,3,2-de]quinoline core. This is exemplified in at least moderate cytotoxicity for all tested compounds containing this moiety, whereas related compounds with an altered core, such as damirones, zyzzyanones and C(16)-C(17)-desaturated pyrroloiminoquinones have generally proven much less active or inactive against mammalian cell lines [7,8,22]. Discorhabdin cytotoxicity has been shown to be affected by the electrophilic reactivity of the spirodienone moiety and correspondingly C-3 carbonyl discorhabdins have proven more cytotoxic than their 3-dihydro analogs or non-electrophilic spiro-derivatives in comparative studies [66,71,73]. Similarly, bromination of the spiro-dienone moiety and C(7)-C(8) desaturation have been shown to increase cytotoxicity against HCT-116, while C-14 bromination, N(18)-C(2) ring closure and substitution of C-1 with bulky substituents are associated with decreased activity [22,72]. The dienone moiety of discorhabdins has been suggested to act as a Michael acceptor in reactions with suitable molecular targets, explaining the effects of C-3 reduction in decreasing cytotoxicity [14].

**Figure 7:**
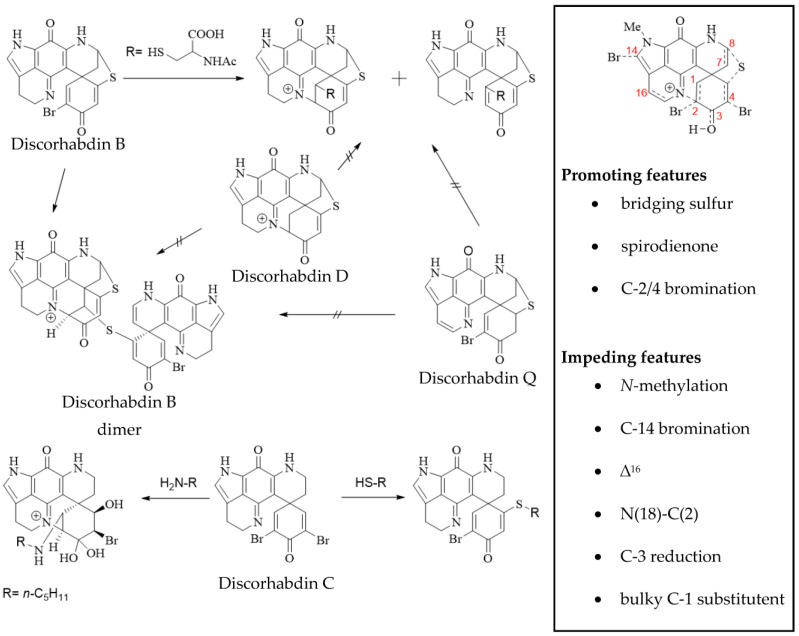
Electrophilic reactivity of discorhabdin B and discorhabdin C (adapted from references [70,71,73]) and structure-activity relationship of discorhabdin cytotoxicity (adapted from references [3,22,71,72]).

Discorhabdin B readily reacts with thiol nucleophiles to yield debrominated C-1 substituted products exhibiting N(18)-C(2) ring closure [70] (Figure 7). The N(18)-C(2) cross-linked discorhabdin D and the 16,17-desaturated discorhabdin Q were found to be unreactive towards nucleophiles, correlating with potent cytotoxicity for discorhabdin B and decreased activity for discorhabdin D and discorhabdin Q. Consequently, this type of nucleophilic addition has been implicated in the mechanism of cytotoxicity for discorhabdins. Recently, discorhabdin C has been reported to react differently with thiol nucleophiles, yielding C-1 or C-5 substituted, monobrominated products with no N(18)-C(2) linkage [73] (Figure 7). However, reaction of discorhabdin C with amine nucleophiles led to a dihydrate product exhibiting N(18)-C(2) ring-closure, while discorhabdin B underwent decomposition at the same conditions. Antitumor in vitro activity was reported to correlate with electrophilic reactivity of the spiro-dienone [73]. Moreover, discorhabdin C can form adducts with the amine-rich protein lysozyme in aqueous medium, suggesting covalent bonding via nucleophilic attack of, e.g., the lysine residues of proteins onto the electrophilic spiro-dienone of C-series discorhabdins [73]. Such reactivity may partly account for the cytotoxic activity of certain discorhabdins and infers a rather unselective mode of action.

**Figure 8:**
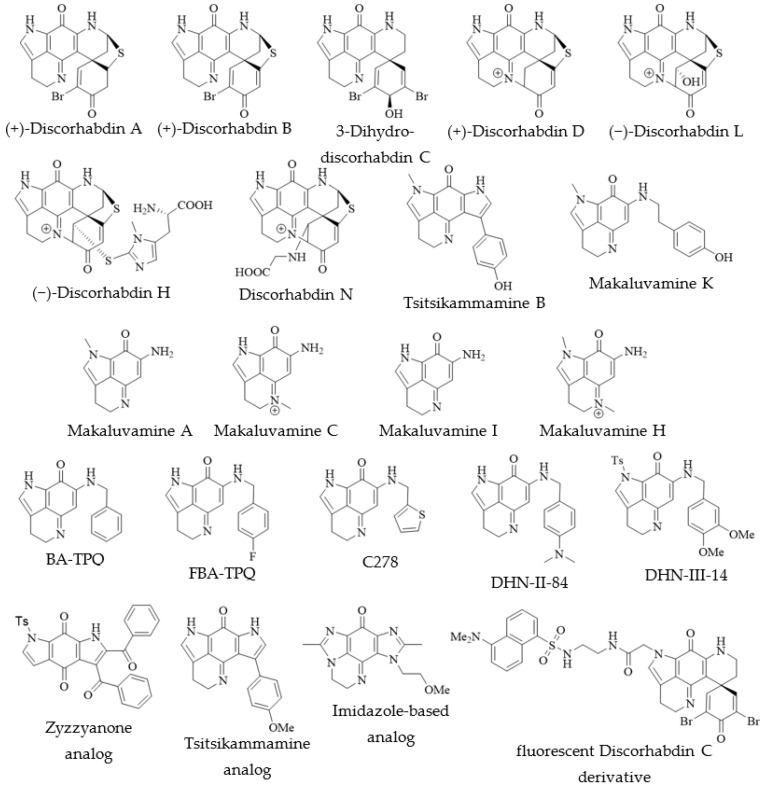
Selected natural and synthetic pyrroloiminoquinones with anticancer potential.

The activity of several natural pyrroloiminoquinones has been evaluated in tumor xenograft models, some returning promising results (Table 1). The first in vivo study of pyrroloiminoquinone bioactivity evaluated the activities of discorhabdin A–C [5]. Despite potent in vitro activity against the P388-cell line, (+)-discorhabdin A (Figure 8) and discorhabdin C (Figure 7) were ineffective in extending the life span of affected model mice and proved toxic to the test animals at 2 mg/kg bodyweight, while (+)-discorhabdin B (Figure 8) effected some tumor reduction. A study published by the same group shortly after evaluated the N(18)-C(2) bridged (+)-discorhabdin D (Figure 8) correspondingly and revealed lower in vitro activity than for the two other compounds, but significant in vivo anti-tumor efficacy [6].

In 1993 Radisky et al. [7] published the first mechanistic investigation into pyrroloiminoquinone cytotoxicity, assessing the anti-cancer potential of several makaluvamines and damirones as well as (+)-discorhabdin A (Figure 8). The compounds were evaluated in cytotoxicity assays against human colon cancer cell line HCT-116, as well as Chinese hamster ovarian (CHO) cell lines xrs-6 and BR1. Additionally, compounds were tested for topoisomerase II inhibition, DNA intercalation and the ability to damage DNA in vitro. Most makaluvamines were found to be moderately to potently cytotoxic. However, (+)-discorhabdin A showed by far the most potent cytotoxicity (IC_50_ = 0.08 µM against HCT-116), while *ortho*-quinones were not active. Cytotoxic compounds were up to nine times less active against the DNA-repair proficient CHO-strain, BR1, than against xrs-6, suggesting a relationship between cytotoxic effects and DNA damage. In addition, the cytotoxic compounds were found to cause single strand cleavage of DNA in vitro upon reductive activation with dithionite at an efficiency corresponding to their ease of reduction and loosely correlating with increasing cytotoxicity [7]. All tested pyrroloiminoquinones proved efficient DNA-intercalators; topoisomerase II inhibition was, however, only observed for makaluvamines. Consequently, topoisomerase II inhibition was suggested as the mechanism of cytotoxicity for makaluvamines, while (+)-discorhabdin A appeared to act via a separate mechanism. Radisky et al. [7] further evaluated makaluvamine A and C (Figure 8) for in vivo anti-cancer activity, finding only marginal life extension in nude mice afflicted by murine leukaemia (P388), but significant reduction of tumor size in human ovarian cancer (OVCAR-3) xenografts in athymic mice (Table 1).

In 2005 Dijoux et al. [8] corroborated the relationship of cytotoxicity and topoisomerase II inhibition for makaluvamines and reported differential cytotoxic activity for several members of this compound class tested in a 60-cell line NCI cancer panel assay. Subsequent in vivo study of makaluvamines H and I (Figure 8) in KB tumor cell xenografts to nude mice (Table 1) revealed that makaluvamine H exhibited better T/C values than the positive control etoposide (38% vs. 40%) at less than half the dose (22 vs. 55 mg/kg). Makaluvamine I proved toxic to the mice at tested doses but still achieved a T/C value of 38% [8]. 

More recently, natural pyrroloiminoquinones have been subject of renewed interest. Goey et al. [21] identified several discorhabdins, including 3-dihydrodiscorhabdin C, (+)-discorhabdin B, (−)-discorhabdin L, (−)-discorhabdin H (Figure 8), as well as makaluvamine F (Figure 2) as potent inhibitors of the complexation of HIF-1α and the coactivator p300. Additionally, some compounds were found to selectively inhibit HIF-1α mediated transcription of a reporter plasmid in HCT-116 and LNCaP at non-toxic concentrations. Furthermore, the secretion of the downstream HIF-1α-mediated vascular endothelial growth factor (VEGF) was found decreased in LNCaP cells treated with (−)-discorhabdin L or (−)-discorhabdin H [21]. HIF-1α is an important transcription factor, involved in regulating cell growth and the response to hypoxia, and elevated levels of HIF-1α are associated with solid tumors and angiogenesis [21]. Importantly, the tested compounds were also found to exhibit much decreased cytotoxicity (IC_50_ = 1.7 to >10 µM against HCT-116) compared with previous reports in the literature [2,3], which characterized most makaluvamines and discorhabdins as potently cytotoxic. This discrepancy was attributed to the comparatively short treatment times and hypoxic conditions used by Goey et al. [21], opposed to longer treatment times and normoxic assay conditions employed by other authors [22].

A follow-up study, investigating the antiangiogenic potential of the VEGF secretion-inhibiting (−)-discorhabdin H and (−)-discorhabdin L, reported low activity against human umbilical vein endothelial cells (HUVEC) for the former compound (IC_50_ > 10 µM) and relatively potent activity for the latter (IC_50_~5 µM), regardless of treatment times (24 and 48 h) or oxygen levels (normoxic and hypoxic) [9]. (−)-Discorhabdin L was also found to inhibit tube formation in HUVEC and strongly decreased micro-vessel outgrowth in an ex vivo mouse aorta ring model. Moreover, this compound stalled prostate cancer (LNCaP) tumor growth in a xenograft model while showing no toxicity to the host mice at the active concentrations (Table 1).

Several groups have employed in silico methods to identify possible cellular targets of pyrroloiminoquinones. Such computational studies have identified discorhabdin N (Figure 8) as a potential allosteric modulator of human heat shock proteins Hsc70 and Hsp72, suspected factors in oncogenesis and cancer procession [103]. Tsitsikammamines as well as A- and D- series discorhabdins have been implicated as potential inhibitors of indoleamine 2,3-dioxygenase (IDO1) and topoisomerase I and II by establishing plausible binding modes through molecular modeling [54,72].

In addition to natural pyrroloiminoquinones synthetic *N*-benzyl and *N*-fluorobenzyl makaluvamine analogs BA-TPQ (*N*-benzyl makaluvamine I) and FBA-TPQ (Figure 8) were discovered as promising anti-cancer drug leads, culminating in preclinical in vivo studies and pharmacological evaluations [99,100,101,104,105,106]. BA-TPQ has been shown to inhibit tumor growth in prostate- and breast cancer xenografts in mice, while not being overtly toxic to the test animals, despite accumulating in their lungs, kidneys and spleens [104,105,106]. The fluoro-benzyl analog FBA-TPQ was found to selectively induce cell cycle arrest and apoptosis in prostate cancer cell lines LNCaP and PC3 in a dose-dependent manner, in addition to reducing the androgen receptor and prostate specific antigen levels [104]. Furthermore, FBA-TPQ inhibited pancreatic- [101], breast- [99] and ovarian [100] tumor growth in mouse xenograft models while exhibiting only minimal toxic effects. The mechanism of action for BA-TPQ has been suggested to involve activation of apoptotic receptors, possibly through formation of a reactive oxygen species [106].

A multitude of other synthetic analogs have been evaluated for bioactivity, including synthetic makaluvamine analogs DHN-II-84 and DHN-III-14 (Figure 8) which decreased c-KIT expression and elicited decreased neuroendocrine tumor markers myeloid cell leukemia-1 (MCL-1), X-chromosome linked inhibitor of apoptosis (XIAP), chromogranin A (CgA) and achaete-scute homolog 1 (ASCL1) [107]. Furthermore, a range of synthetic wakayin, tsitsikammamine and zyzzyanone analogs were tested in cellular assays of HEK 293-EBNA cell lines expressing hIDO1 or hTDO [108]. Interestingly, a zyzzyanone analog (Figure 8), which is not strictly speaking a pyrroloiminoquinone, showed the most promising inhibitory profile with ca. 52% (IDO) and 15% (TDO) inhibition at 3.12 µM concentration, while exhibiting virtually no effect on cell viability [108]. In yet another study, a set of pyrroloiminoquinone analogs was synthesized to investigate their potential as anti-skin cancer drug leads [109]. Compound C278 (Figure 8) returned the most favorable results, being two-fold active against skin cancer SCC13 cells over normal human keratinocyte HaCaT cells (IC_90_ = 0.9 vs. 2.1 µM) and displaying dose-dependent inhibition of SCC13 cell migration and invasion as well as eliciting apoptosis [109]. In addition, synthetic tsitsikammamine analogs have shown promising sub-micromolar inhibitory activity against IDO1 in an enzyme assay, while retaining some activity in cell-based assays [110]. Meanwhile, the most active analog (Figure 8) showed neither cell-based TDO inhibitory activity nor any inhibition of cell viability at 10 µM concentration [110]. Hoang et al. [111] synthesized a range of imidazole-based pyrroloiminoquinone analogs for evaluation of cytotoxic and cytostatic effects in a 60-cancer cell panel assay. A *N-*(methoxy)ethyl-substituted analog (Figure 8) showed the most promising cytostatic activity against A498 (renal cancer cell line), being four orders of magnitude more active than its’ *N*-methyl analog. Correlation analysis using the NCI COMPARE algorithm identified the VEGF (Flt-1) receptor as a potential target [111]. Lastly, Lam et al. [112] developed a protocol of modifying pyrroloiminoquinones with fluorescent probes for potential cell localization studies and identification of protein targets. One example was the reaction of discorhabdin C with an ethylenediamine linker, followed by addition of a dansyl fluorophore, where the final product showed comparable cytotoxic activity to the natural parent compound (IC_50_ against P388 = 0.34 vs. 0.11 µM).

Taken together, the results discussed in this section highlight that the cytotoxic mode of action of the pyrroloiminoquinones is still not well understood and likely involves multiple concurring and structure-dependent mechanisms, warranting further study to unravel the biological effects of these diverse natural products. The cytotoxicity of makaluvamines is likely mediated through topoisomerase II inhibition [7,8], while discorhabdin cytotoxicity is promoted by increasing electrophilic reactivity of their spiro-dienone moiety, which also promotes nonselective toxicity [70,71,73]. Interestingly, the most promising in vivo anticancer results in the literature have been reported for pyrroloiminoquinones that do not possess the reactive combination of a nucleophilic N(18) and a electrophilic spiro-dienone moiety, such as makaluvamines H and I [8], discorhabdin D [6] and discorhabdin L [9]. It is tempting therefore to hypothesize that high electrophilic reactivity of the dienone-moiety may result in nonselective reaction with a multitude of cellular targets, masking more selective action, e.g. HIF-1 α inhibition, which may come to prevail in those pyrroloiminoquinones that bear a less reactive dienone-moiety.

**Figure 9:**
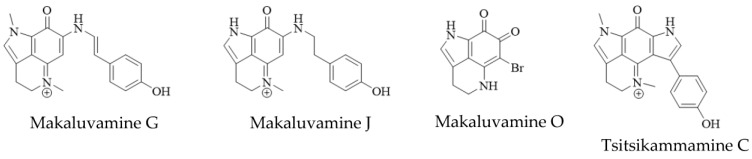
Selected pyrroloiminoquinones and related compounds with antiplasmodial potential.

Several pyrroloiminoquinones have been shown to exhibit similar magnitudes of cytotoxicity against human cancer and non-cancer cell lines; therefore, pyrroloiminoquinones cannot summarily be described as exhibiting selective cytotoxicity against human cancer cell lines over human non-cancer cell lines. Nevertheless, comparison of literature reports yielded notable exceptions that may exhibit selective activity against cancer cell lines, including makaluvamine J and K as well as tsitsikammamine B. For instance, makaluvamine J (Figure 9) and K (Figure 8) showed IC_50_ values of 0.054 and 0.056 µM against PANC1, respectively, [46] as opposed to reported IC_50_ values of 1.2 and 1.1 µM against HEK293 [10], whereas tsitsikammamine B (Figure 8) was potently active against HCT-116 (IC_50_ = 0.222 µM) [22] while only showing weak activity against HEK293 at 50 µM concentration [49].

### 4.2. Antiplasmodial Potential

A few pyrroloiminoquinones of multiple classes have been identified as possible antimalarial lead compounds. Makaluvamine O (Gordon and Betty Moore Foundation), which has been shown to be inactive for inhibiting viability of HEK293 cells [49], exhibits moderate in vitro activity against the chloroquine-sensitive D6 clone of *Plasmodium falciparum* (IC_50_ = 0.94 µg/mL) with five-fold selectivity over Vero cells [89]. Potent anti-plasmodial activity was reported for (+)-discorhabdin A (Figure 8) against chloroquine-sensitive (D6 clone) and chloroquine-resistant (W2 clone) strains of *P. falciparum* (IC_50_ = 53 nM for both) [12]. Interestingly, 3-dihydrodiscorhabdin C (Figure 8) showed some antiplasmodial activity (IC_50_ = 170 nM vs. D6, IC_50_ = 130 nM vs. W2) whereas discorhabdin C (Figure 7) was least active (IC_50_ = 2800 nM vs. D6, IC_50_ = 2000 nM vs. W2), despite bearing the reactive α-bromoenone moiety that promotes mammalian cell cytotoxicity. Particularly, (+)-discorhabdin A and 3-dihydrodiscorhabdin C were significantly less active against murine Vero cells with IC_50_-values in the low micromolar range, providing evidence for selectivity towards *P. falciparum* [12].

The most active anti-plasmodial compounds, (+)-discorhabdin A and 3-dihydrodiscorhabdin C, were chosen for in vivo evaluation in *Plasmodium berghei*-infected mice [12]. Unfortunately, both compounds had severe cytotoxic effects on the mice and all animals treated with 3-dihydrodiscorhabdin C died before investigation of parasitemia. Discorhabdin A proved slightly less toxic to the host animals, with 4 of 5 animals losing weight and showing severe intoxication but surviving with parasitemia found reduced by 50% [12].

Evaluation of the antimalarial potential of several makaluvamines and damirones as well as tsitsikammamine C (Figure 9) isolated from an Australian *Zyzzya* sp. sponge [10] showed that makaluvamines inhibit chloroquine-resistant and chloroquine-sensitive strains of *P. falciparum* with IC_50_-values in the mid-nanomolar range opposed to micromolar activities against HEK293 cells. The most active compound tested and with the highest selectivity index, was the bispyrroloiminoquinone tsitsikammamine C, which was excluded from subsequent in vivo evaluation due to a paucity of material. Nonetheless, makaluvamine J and makaluvamine G (Figure 9) were further tested against *P. berghei* in a mouse model. Makaluvamine G showed promising anti-plasmodial activity, suppressing *P. berghei* infection more effectively than the positive control chloroquine (48% vs. 35% on 4th day post infection), while makaluvamine J showed no antiplasmodial in vivo effect and proved toxic to the test animals [10].

Recently, Lam et al. [73] reported antiprotozoal activity for several discorhabdins of the A-, D- and C-series and some semi-synthetic derivatives. Several compounds showed potent inhibitory activities, including (-)-discorhabdin L (Figure 8) which returned an IC_50_-value of 0.03 µM against *P. falciparum* (K1 strain) vs. 1.1 µM against rat skeletal myoblast cell line L6. The most selective activity was however observed for the thioether-linked discorhabdin B dimer (Figure 3), which exhibited IC_50_ values of 0.08 and 41 µM against *P. falciparum* and non-malignant rat skeletal myoblast L6 cells, respectively. Noteworthy, this dimer has shown potent cytotoxicity against HCT-116 cells in previous works [74] and it is unclear what underlies the pronounced difference in activity against these two cancer cell lines. Overall, antimalarial activity was not found to correlate with the electrophilic reactivity (and hence antimammalian cytotoxicity) of the spiro-dienone arrangement in discorhabdins, which may suggest a specific mode of action of pyrroloiminoquinones on a yet unknown cellular target in protozoa.

### 4.3. Antimicrobial, Antifungal, Neuromodulatory and Antioxidant Potential

**Figure 10:**
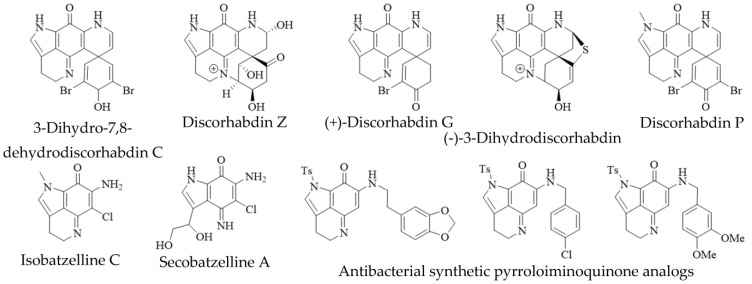
Selected natural and synthetic pyrroloiminoquinones with antibacterial, antiviral, antifungal or neuromodulatory potential.

In addition to mammalian and protozoal cytotoxicity, pyrroloiminoquinones have been reported to exhibit antifungal, antibacterial, antiviral, neuromodulatory and antioxidant activities. For instance, makaluvamines, tsitsikammamines and discorhabdins have been reported as antibacterial agents against several Gram-(+) and Gram-(−) bacteria [5,12,15,16,39,62,113]. Most antibacterial evaluations were carried out using zone-inhibition or not further specified assays. Nevertheless, Jeon et al. [16] established MIC values for several discorhabdins and makaluvamines against three Gram-(+) (*Bacillus subtilis*, *Micrococcus luteus*, *Staphylococcus aureus*) and three Gram-(−) (*Escherichia coli*, *Proteus vulgaris*, *Salmonella typhimurium*) bacteria. None of the tested compounds showed significant activity against *E. coli* (MIC > 100 µM) and the most potent activities were exhibited by (+)-discorhabdin D (6.25 µM, Figure 8) and makaluvamine F (3.125 µM, Figure 2) against *M. luteus*, as well as by (+)-discorhabdin B (Figure 8) against *P. vulgaris* (3.125 µM). Na et al. [12] provided evidence for antimicrobial activity of (+)-discorhabdin A (Figure 8), discorhabdin C (Figure 7) and 3-dihydrodiscorhabdin C (Figure 8) against methicillin-resistant *S. aureus* (MRSA), *Mycobacterium intracellulare* and *Mycobacterium tuberculosis*. (+)-Discorhabdin A inhibited viability of MRSA and *M. intracellulare* with IC_50_ values of 4.8 µM (MIC = 12 µM) and 0.46 µM (MIC = 0.74 µM), respectively, while for inhibition of *M. tuberculosis* only an MIC value of 7.7 µM was reported. Discorhabdin C also proved remarkable activity against all three bacteria (MRSA- IC_50_/MIC = 3.2/11 µM; *M. intracellulare*- 0.13/0.17 µM; *M. tuberculosis*- 6.8/8.0 µM), while 3-dihydrodiscorhabdin was less active against MRSA (IC_50_ = 13 µM) and *M. tuberculosis* (MIC = 14 µM) and inactive against *M. intracellulare*. In addition, discorhabdin Z and (−)-3-dihydrodiscorhabdin D (Figure 10) showed the ability to inhibit sortase A, an enzyme involved in bacterial cell-adhesion [16]. Furthermore, Nijampatnam et al. [114] synthesized 14 makaluvamine analogs with antibiotic activity against *Streptococcus mutans*, as well as the ability to inhibit *S. mutans* biofilm formation. Three compounds (Figure 10) were found to inhibit biofilm formation with IC_50_ values considerably lower than MIC_50_ values for bactericidal activity, suggesting these compounds may exhibit antibiofilm activities rather than simply killing the responsible bacteria [114].

The antiviral activity of pyrroloiminoquinones has only been superficially investigated. Noteworthy, discorhabdins were originally discovered from crude extracts prioritized due to their antiviral in vitro activity [5], yet the first published assay results were only provided in 2002 [11]. These authors evaluated the ability of several isobatzellines, batzellines and makaluvamines to inhibit HIV-1 envelope mediated cell-fusion in a β-galactose reporter assay. While particularly low reporter activities were observed with isobatzelline C (Figure 10), followed by makaluvamine A and H (Figure 8), the authors of this study suggested that the activity in this assay may have resulted from inhibition of DNA-modifying enzymes, since the results correlated with literature reports of topoisomerase II inhibition [11]. Another report concerning antiviral activity of pyrroloiminoquinones was provided for (+)-discorhabdin A, discorhabdin C and 3-dihydrodiscorhabdin C which inhibit the proliferation of the HCV-replicon in human cell culture (Huh7), but also proved cytotoxic to the host cells [12].

In addition to the activities discussed above, (+)-discorhabdin G, 3-dihydro-7,8-dehydrodiscorhabdin C (Figure 10), (+)-discorhabdin B (Figure 8) and (−)-discorhabdin L (Figure 8) have been shown to exhibit competitive reversible inhibition of electric eel (eeAChE) and recombinant human acetylcholinesterase (hAChE), as well as horse serum butyrylcholinesterase (BChE) [18]. (+)-Discorhabdin B proved the most potent inhibitory activity against hAChE among tested compounds (22.8 µM). The most active compound against eeAChE and BChE, (+)-discorhabdin G (IC_50_ = 1.6 and 7 µM, respectively), was found to show no undesirable electrophysiological effects after further testing. Interactions between the test compounds and actives sites of the enzymes were defined through in silico molecular docking which returned binding energies correlating with the in vitro results. It was suggested that the discorhabdin scaffold may represent a valuable pharmacophore for the development of drugs to treat dementia, Alzheimer’s disease and other neurological disorders associated with elevated cholinesterase activity [18]. Furthermore, makaluvamine G (Figure 9) has been identified as an inhibitor of the muscle nicotinic acetylcholine receptor [47]. Several makaluvamines have been tested for antioxidant activities, finding makaluvamine J (Figure 9) most active in reducing mitochondrial damage by H_2_O_2_ [20]. This activity was ascribed to be in part due to an elicited improvement in the endogenous antioxidant defenses of glutathione and catalase [20]. Lastly, discorhabdin P (Figure 10) was shown to inhibit the phosphatase activity of calcineurin with an IC_50_ value of 0.55 µg/mL [61] with secobatzelline A (Figure 10) showing equally potent activity [19]. Both compounds also inhibited the peptidase activity of CPP32 with 0.37 µg/mL and 0.02 µg/mL, respectively, while discorhabdin C showed no inhibitory activity against either enzyme [61].

To our knowledge no studies have investigated the antibacterial or antifungal potential of damirones, nor the antiviral potential of bispyrroloiminoquinones. Furthermore, bispyrroloiminoquinones have not been evaluated for anticancer or antimalarial activity in vivo, despite sometimes promising in vitro results. In addition, unusual pyrroloiminoquinones and discorhabdin oligomers have only been very sparsely researched concerning their biological activities.

## 5. Conclusions

Pyrroloiminoquinone natural products are widely distributed across diverse organisms from marine as well as terrestrial sources and display an immense structural diversity. Their biological function includes feeding deterrence [115] but is not fully understood and these compounds may also play an immunological role in protection against pathogens and mediation of the sponge microbiome, considering their antimicrobial and enzyme inhibitory activities. Currently, the biosynthesis of pyrroloiminquinones is largely based on logical deduction with only little experimental validation and the responsible biosynthetic gene clusters are still unknown. The pyrroloiminoquinone core as exemplified in unbranched makaluvamines is not restricted to marine sponges, but also found in ascidians, myxomycetes and hydroids, while closely related natural products are present in marine-derived actinobacteria and terrestrial fungi. Discorhabdins are produced only by sponges, while bispyrroloiminoquinones are limited to marine sponges and ascidians. Whether or not the biosynthesis of these diverse compounds is unified by a common pathway remains to be discovered.

Pyrroloiminoquinones exhibit a broad range of potent bioactivities, including selective antitumor cytotoxicity, antimicrobial activity, antimalarial activity, as well as antifungal and antiviral activities. Several natural and synthetic pyrroloiminoquinones have produced promising results in preclinical in vivo antitumor and antiplasmodial assays, suggesting potential for further development of new drugs in this regard based on the structural motif of pyrroloiminoquinones. To date, synthetic analogs, rather than pyrroloiminoquinone natural products, are most advanced in preclinical evaluation, yielding promising results against several cancer cell lines, including evidence for in vivo efficacy. By contrast, the antimicrobial activity of pyrroloiminoquinones has not been thoroughly researched and, as of yet, only limited mechanistic investigations have been carried out. Antimalarial application of pyrroloiminoquinones has not been patented, despite promising results for a range of pyrroloiminoquinones, while their antifungal and antiviral potential has only been superficially investigated.

Attenuated electrophilic activity may play an important role in increasing cytotoxic selectivity, particularly for discorhabdins, and may provide an avenue to make pyrroloiminoquinones more amenable to pharmaceutical utilization. Furthermore, pyrroloiminoquinones could prove to be valuable warheads in warhead-carrier assemblies and unselective cytotoxicity of some pyrroloiminoquinones may be overcome by employing targeted drug delivery, such as through nanocarrier delivery systems. Given their great inhibitory potential towards a variety of targets, further investigations of pyrroloiminoquinone biological activities are warranted and their activity in compositional drug delivery systems should be investigated.

Finally, the amenability to chemical modification and broad spectrum of activities and potential targets make pyrroloiminoquinones attractive chemical probes for the development of new therapeutics. However, their broad and potent cytotoxicity will present challenges for their progression from the laboratory and into the drug discovery pipeline.

## Figures and Tables

**Table 1:** Anti-tumor activity of natural pyrroloiminoquinones against tumor xenografts in mouse models.

Compound	Cell Line	Activity	Dose	Reference
(+)-Discorhabdin A	P-388	Toxic	2 mg/kg	[5]
(+)-Discorhabdin B	P-388	T/C(life span) = 117%	0.25 mg/kg	[5]
Discorhabdin C	P-388	Toxic	2 mg/kg	[5]
(+)-Discorhabdin D	P-388	T/C(life span) = 132%	20 mg/kg	[6]
(−)-Discorhabdin H	LNCaP	T/C(tumor size) = 100%	5 mg/kg	[9]
(−)-Discorhabdin L	LNCaP	T/C(tumor size) ≈ 50%	5 mg/kg	[9]
Makaluvamine A	OVCAR-3/P-388	ILS = 0%/T/C(tumor size) = 62%	0.5 mg/kg	[7]
Makaluvamine C	OVCAR-3/P-388	ILS = 18%/T/C(tumor size) = 48%	5.0 mg/kg	[7]
Makaluvamine H	KB	T/C(tumor size) = 38%	22 mg/kg	[8]
Makaluvamine I	KB	T/C(tumor size) = 34%, toxic	22 mg/kg	[8]

T/C denotes the response ratio between test and control animal. P-388 murine leukaemia, LNCaP metastatic prostate cancer, OVCAR-3 ovarian cancer, KB skin and cervical cancer.

## Data Availability

Not applicable.

## References

[1] Urban S., Hickford S.J.H., Blunt J.W., Munro M.H.G. (2000). Bioactive marine alkaloids. Curr. Org. Chem..

[2] Antunes E.M., Copp B.R., Davies-Coleman M.T., Samaai T. (2005). Pyrroloiminoquinone and related metabolites from marine sponges. Nat. Prod. Rep..

[3] Hu J., Fan H., Xiong J., Wu S. (2011). Discorhabdins and pyrroloiminoquinone-related alkaloids. Chem. Rev..

[4] Li F., Kelly M., Tasdemir D. (2021). Chemistry, Chemotaxonomy and Biological Activity of the Latrunculid Sponges (Order Poecilosclerida, Family Latrunculiidae). Mar. drugs.

[5] Perry N.B., Blunt J.W., Munro M.H.G. (1988). Cytotoxic pigments from New Zealand sponges of the genus *Latrunculia*: Discorhabdins A, B and C. Tetrahedron.

[6] Perry N.B., Blunt J.W., Munro M.H.G. (1988). Discorhabdin D, an Antitumor Alkaloid from the Sponges *Latrunculia brevis* and *Prianos* sp.. J. Org. Chem..

[7] Radisky D.C., Radisky E.S., Barrows L.R., Copp B.R., Kramer R.A., Ireland C.M. (1993). Novel cytotoxic topoisomerase II inhibiting pyrroloiminoquinones from Fijian sponges of the genus *Zyzzya*. J. Am. Chem. Soc..

[8] Dijoux M.-G., Schnabel P.C., Hallock Y.F., Boswell J.L., Johnson T.R., Wilson J.A., Ireland C.M., van Soest R., Boyd M.R., Barrows L.R. (2005). Antitumor activity and distribution of pyrroloiminoquinones in the sponge genus *Zyzzya*. Bioorg. Med. Chem..

[9] Harris E.M., Strope J.D., Beedie S.L., Huang P.A., Goey A.K.L., Cook K.M., Schofield C.J., Chau C.H., Cadelis M.M., Copp B.R. (2018). Preclinical evaluation of discorhabdins in antiangiogenic and antitumor models. Mar. Drugs.

[10] Davis R.A., Buchanan M.S., Duffy S., Avery V.M., Charman S.A., Charman W.N., White K.L., Shackleford D.M., Edstein M.D., Andrews K.T. (2012). Antimalarial activity of pyrroloiminoquinones from the Australian marine sponge *Zyzzya* sp.. J. Med. Chem..

[11] Chang L.C., Otero-Quintero S., Hooper J.N.A., Bewley C.A. (2002). Batzelline D and Isobatzelline E from the Indopacific Sponge *Zyzzya fuliginosa*. J. Nat. Prod..

[12] Na M., Ding Y., Wang B., Tekwani B.L., Schinazi R.F., Franzblau S., Kelly M., Stone R., Li X.-C., Ferreira D. (2010). Anti-infective Discorhabdins from a Deep-Water Alaskan Sponge of the Genus *Latrunculia*. J. Nat. Prod..

[13] Sun H.H., Sakemi S., Burres N., McCarthy P. (1990). Isobatzelline A, B, C and D. Cytotoxic and antifungal pyrroloquinoline alkaloids from the marine sponge *Batzella* sp.. J. Org. Chem..

[14] Copp B.R., Fulton K.F., Perry N.B., Blunt J.W., Munri M.H.G. (1994). Natural and Synthetic Derivatives of Discorhabdin C, a Cytotoxic Pigment from the New Zealand Sponge *Latrunculia* cf. bocagei. J. Org. Chem..

[15] Hooper G.J., Davies-Coleman M.T., Kelly-Borges M., Coetzee P.S. (1996). New alkaloids from a South African latrunculid sponge. Tetrahedron Lett..

[16] Jeon J., Na Z., Jung M., Lee H., Sim C.J., Nahm K., Oh K.-B., Shin J. (2010). Discorhabdins from the Korean marine sponge *Sceptrella* sp.. J. Nat. Prod..

[17] Crews P., Valeriote F.A., Lin S., McCauley E.P., Lorig-Roach N., Tenney K. (2021). Pyrroloquinolin Compounds and Methods of Using Same. U.S. patent.

[18] Botić T., Defant A., Zanini P., Žužek M.C., Frangež R., Janussen D., Kersken D., Knez Ž., Mancini I., Sepĉić K. (2017). Discorhabdin alkaloids from Antarctic *Latrunculia* spp. sponges as a new class of cholinesterase inhibitors. Eur. J. Med. Chem..

[19] Gunasekera S.P., McCarthy P.J., Longley R.E., Pomponi S.A., Wright A.E. (1999). Secobatzellines A and B, Two New Enzyme Inhibitors from a Deep-Water Caribbean Sponge of the Genus *Batzella*. J. Nat. Prod..

[20] Alonso E., Alvariño R., Leirós M., Tabudravu J.N., Feussner K., Dam M.A., Rateb M.E., Jaspars M., Botana L.M. (2016). Evaluation of the antioxidant activity of the marine pyrroloiminoquinone makaluvamines. Mar. Drugs.

[21] Goey A.K.L., Chau C.H., Sissung T.M., Cook K.M., Venzon D.J., Castro A., Ransom T.R., Henrich C.J., McKee T.C., McMahon J.B. (2016). Screening and biological effects of marine pyrroloiminoquinone alkaloids: Potential inhibitors of the HIF-1α/p300 interaction. J. Nat. Prod..

[22] Antunes E.M., Beukes D.R., Kelly M., Samaai T., Barrows L.R., Marshall K.M., Sincich C., Davies-Coleman M.T. (2004). Cytotoxic pyrroloiminoquinones from four new species of South African latrunculid sponges. J. Nat. Prod..

[23] Copp B.R., Ireland C.M. (1991). Wakayin: A Novel Cytotoxic Pyrroloiminoquinone Alkaloid from the Ascidian Clavelina Species. J. Org. Chem..

[24] Grkovic T., Ruchirawat S., Kittakoop P., Grothaus P.G., Evans J.R., Britt J.R., Newman D.J., Mahidol C., O’Keefe B.R. (2021). A New Bispyrroloiminoquinone Alkaloid From a Thai Collection of *Clavelina* sp.. Asian J. Org. Chem..

[25] Ishibashi M., Iwasaki T., Imai S., Sakamoto S., Yamaguchi K., Ito A. (2001). Laboratory culture of the myxomycetes: Formation of fruiting bodies of Didymium bahiense and its plasmodial production of makaluvamine A. J. Nat. Prod..

[26] Nakatani S., Kiyota M., Matsumoto J., Ishibashi M. (2005). Pyrroloiminoquinone pigments from Didymium iridis. Biochem. Syst. Ecol..

[27] Zlotkowski K., Hewitt W.M., Yan P., Bokesch H.R., Peach M.L., Nicklaus M.C., O’Keefe B.R., McMahon J.B., Gustafson K.R., Schneekloth J.S. (2017). Macrophilone A: Structure Elucidation, Total Synthesis, and Functional Evaluation of a Biologically Active Iminoquinone from the Marine Hydroid *Macrorhynchia philippina*. Org. Lett..

[28] Yan P., Ritt D.A., Zlotkowski K., Bokesch H.R., Reinhold W.C., Schneekloth J.S., Morrison D.K., Gustafson K.R. (2018). Macrophilones from the Marine Hydroid *Macrorhynchia philippina* Can Inhibit ERK Cascade Signaling. J. Nat. Prod..

[29] Peters S., Spiteller P. (2007). Sanguinones A and B, Blue Pyrroloquinoline Alkaloids from the Fruiting Bodies of the Mushroom *Mycena sanguinolenta*. J. Nat. Prod..

[30] Peters S., Jaeger R.J.R., Spiteller P. (2008). Red Pyrroloquinoline Alkaloids from the Mushroom *Mycena haematopus*. Eur. J. Org. Chem..

[31] Pulte A., Wagner S., Kogler H., Spiteller P. (2016). Pelianthinarubins A and B, Red Pyrroloquinoline Alkaloids from the Fruiting Bodies of the Mushroom *Mycena pelianthina*. J. Nat. Prod..

[32] Lohmann J.S., Wagner S., von Nussbaum M., Pulte A., Steglich W., Spiteller P. (2018). Mycenaflavin A, B, C and D: Pyrroloquinoline Alkaloids from the Fruiting Bodies of the Mushroom *Mycena haematopus*. Chem. Eur. J..

[33] Nagata H., Ochiai K., Aotani Y., Ando K., Yoshida M., Takahashi I., Tamaoki T. (1997). Lymphostin (LK6-A), a Novel Immunosuppressant from *Streptomyces* sp. KY11783: Taxonomy of the Producing Organism, Fermentation, Isolation, and Biological Activities. J. Antibiot..

[34] Hughes C.C., MacMillan J.B., Gaudencio S.P., Jensen P.R., Fenical W. (2009). The ammosamides: Structures of cell cycle modulators from a marine-derived *Streptomyces* species. Angew. Chem. Int. Ed..

[35] Harayama Y., Kita Y. (2005). Pyrroloiminoquinone Alkaloids: Discorhabdins and Makaluvamines. Curr. Org. Chem..

[36] Wada Y., Fujioka H., Kita Y. (2010). Synthesis of the Marine Pyrroloiminoquinone Alkaloids, Discorhabdins. Mar. Drugs.

[37] Smith M.W., Falk I.D., Ikemoto H., Burns N.Z. (2019). A convenient C–H functionalization platform for pyrroloiminoquinone alkaloid synthesis. Tetrahedron.

[38] Carney J.R., Scheuer P.J., Kelly-Borges M. (1993). Makaluvamine G, a cytotoxic pigment from an Indonesian sponge *Histodermella* sp.. Tetrahedron.

[39] Schmidt E.W., Harper M.K., Faulkner D.J. (1995). Makaluvamines H-M and damirone C from the Pohnpeian sponge *Zyzzya fuliginosa*. J. Nat. Prod..

[40] Fu X., Ng P.-L., Schmitz F.J., Hossain M.B., van der Helm D., Kelly-Borges M. (1996). Makaluvic acids A and B: Novel Alkaloids from the Marine Sponge *Zyzzya fuliginosus*. J. Nat. Prod..

[41] Venables D.A., Concepciόn G.P., Matsumoto S.S., Barrows L.R., Ireland C.M. (1997). Makaluvamine N: A new pyrroloiminoquinone from *Zyzzya fuliginosa*. J. Nat. Prod..

[42] Popov A.M., Utkina N.K. (1998). Pyrroloquinoline alkaloids from *Zyzzya* sp. sea sponges: Isolation and antitumor activity characterization. Pharm. Chem. J..

[43] Tasdemir D., Mangalindan G.C., Concepción G.P., Harper M.K., Ireland C.M. (2001). 3,7-Dimethylguanine, a New Purine from a Philippine Sponge *Zyzzya fuliginosa*. Chem. Pharm. Bull..

[44] Casapullo A., Cutignano A., Bruno I., Bifulco G., Debitus C., Gomez-Paloma L., Riccio R. (2001). Makaluvamine P, a New Cytotoxic Pyrroloiminoquinone from *Zyzzya* cf. *fuliginosa*. J. Nat. Prod..

[45] Utkina N.K., Makarchenko A.E., Denisenko V.A., Dmitrenok P.S. (2004). Zyzzyanone A, a novel pyrrolo[3,2-f]indole alkaloid from the Australian marine sponge *Zyzzya fuliginosa*. Tetrahedron Lett..

[46] Lin S., McCauley E.P., Lorig-Roach N., Tenney K., Naphen C.N., Yang A., Johnson T.A., Hernandez T., Rattan R., Valeriote F.A. (2017). Another look at pyrroloiminoquinone alkaloids-perspectives on their therapeutic potential from known structures and semisynthetic analogues. Mar. Drugs.

[47] Kudryavtsev D.S., Spirova E.N., Shelukhina I.V., Son L.V., Makarova Y.V., Utkina N.K., Kasheverov I.E., Tsetlin V.I. (2018). Makaluvamine G from the Marine Sponge *Zyzzia fuliginosa* Inhibits Muscle nAChR by Binding at the Orthosteric and Allosteric Sites. Mar. Drugs.

[48] Keyzers R.A., Samaai T., Davies-Coleman M.T. (2004). Novel purroloquinoline ribosides from the South African latrunculid sponge *Strongylodesma aliwaliensis*. Tetrahedron Lett..

[49] Kalinski J.-C.J., Waterworth S.C., Siwe Noundou X., Jiwaji M., Parker-Nance S., Krause R.W.M., McPhail K.L., Dorrington R.A. (2019). Molecular Networking Reveals Two Distinct Chemotypes in Pyrroloiminoquinone-Producing *Tsitsikamma favus* Sponges. Mar. Drugs.

[50] Lill R.E., Major D.A., Blunt J.W., Munro M.H.G., Battershill C.N., McLean M.G., Baxter R.L. (1995). Studies on the biosynthesis of discorhabdin B in the New Zealand sponge *Latrunculia* sp.. J. Nat. Prod..

[51] Parker-Nance S., Hilliar S., Waterworth S., Walmsley T., Dorrington R. (2019). New species in the sponge genus *Tsitsikamma* (*Poecilosclerida*, *Latrunculiidae*) from South Africa. Zookeys.

[52] Kalinski J.C.J., Krause R.W., Parker-Nance S., Waterworth S.C., Dorrington R.A. (2021). Unlocking the diversity of pyrroloiminoquinones produced by Latrunculid sponge species. Mar. Drugs.

[53] Taufa T., Gordon R.M.A., Ali Hashmi M., Hira K., Miller J.H., Lein M., Fromont J., Northcote P.T., Keyzers R.A. (2019). Pyrroloquinoline derivatives from a Tongan specimen of the marine sponge *Strongylodesma tongaensis*. Tetrahedron Lett..

[54] Li F., Pfeifer C., Pérez-Victoria I., Tasdemir D. (2018). Targeted isolation of tsitsikammamines from the Antarctic deep-sea sponge *Latrunculia biformis* by molecular networking and anticancer activity. Mar. Drugs.

[55] D’Ambrosio M., Guerreiro A., Chiasera G., Pietra F. (1996). Epinardins A-D, new pyrroloiminoquinone alkaloids of undetermined deep-water green demosponges from pre-Antarctic Indian Ocean. Tetrahedron.

[56] Kobayashi J., Cheng J.-F., Ishibashi M., Nakamura H., Ohizumi Y., Hirata Y., Sasaki T., Lu H., Clardy J. (1987). Prianosin A, A Novel Antileukemic Alkaloid from the Okinawan Marine Sponge *Prianos melanos*. Tetrahedron Lett..

[57] Cheng J.-F., Ohizumi Y., Wälchli M.R., Nakamura H., Hirata Y., Sasaki T., Kobayashi J. (1988). Prianosins B,C, and D, novel sulfur-containing alkaloids with potent antineoplastic activity from the Okinawan marine sponge *Prianos melanos*. J. Org. Chem..

[58] Perry N.B., Blunt J.W., McCombs J.D., Munro M.H.G. (1986). Discorhabdin C, a highly cytotoxic pigment from a sponge of the genus *Latrunculia*. J. Org. Chem..

[59] Yang A., Baker B.J., Grimwade J., Leonard A., McClintock J.B. (1995). Discorhabdin alkaloids from the Antarctic sponge *Latrunculia apicalis*. J. Nat. Prod..

[60] Dijoux M.-G., Gamble W.R., Hallock Y.F., Cardellina J.H., van Soest R., Boyd M.R. (1999). A New Discorhabdin from Two Sponge Genera. J. Nat. Prod..

[61] Gunasekera S.P., McCarthy P.J., Longley R.E., Pomponi S.A., Wright A.E., Lobkovsky E., Clardy J.J. (1999). Discorhabdin P, a new enzyme inhibitor from deep-water Caribbean sponge of the genus *Batzella*. J. Nat. Prod..

[62] Ford J., Capon R.J. (2000). Disocrhabdin R: A new antibacterial pyrroloiminoquinone from two latrunculid marine sponges, *Latrunculia* sp. and *Negombata* sp.. J. Nat. Prod..

[63] Reyes F., Martín R., Rueda A., Fernández R., Montalvo D., Gómez C., Sánchez-Puelles J.M. (2004). Discorhabdins I and L, Cytotoxic Alkaloids from the Sponge *Latrunculia brevis*. J. Nat. Prod..

[64] Lang G., Pinkert A., Blunt J.W., Munro M.H.G. (2005). Discorhabdin W, the First Dimeric Discorhabdin. J. Nat. Prod..

[65] Grkovic T., Ding Y., Li X.-C., Webb V.L., Ferreira D., Copp B.R. (2008). Enantiomeric Discorhabdin Alkaloids and Establishment of Their Absolute Configurations Using Theoretical Calculations of Electronic Circular Dichroism Spectra. J. Org. Chem..

[66] Grkovic T., Copp B.R. (2009). New natural products in the discorhabdin A- and B-series from New Zealand-sourced *Latrunculia* spp. sponges. Tetrahedron.

[67] El-Naggar M., Capon R.J. (2009). Discorhabdins Revisited: Cytotoxic Alkaloids from Southern Australian Marine Sponges of the Genera Higginsia and Spongosorites. J. Nat. Prod..

[68] Grkovic T., Pearce A.N., Munro M.H.G., Blunt J.W., Davies-Coleman M.T., Copp B.R. (2010). Isolation and Characterization of Diastereomers of Discorhabdins H and K and Assignment of Absolute Configuration to Discorhabdins D, N, Q, S, T, and U. J. Nat. Prod..

[69] Makar’eva T.N., Krasokhin V.B., Guzii A.G., Stonik V.A. (2010). Strong ethanol solvate of discorhabdin A isolated from the far-east sponge *Latruculia oparinae*. Chem. Nat. Comp..

[70] Lam C.F.C., Grkovic T., Pearce N.A., Copp B.R. (2012). Investigation of the electrophilic reactivity of the cytotoxic marine alkaloid discorhabdin B. Org. Biomol. Chem..

[71] Lam C.F.C., Cadelis M.M., Copp B.R. (2017). Exploration of the influence of spiro-dienone moiety on biological activity of cytotoxic marine alkaloid discorhabdin P. Tetrahedron.

[72] Li F., Peifer C., Janussen D., Tasdemir D. (2019). New Discorhabdin Alkaloids from the Antarctic Deep-Sea Sponge *Latrunculia biformis*. Mar. Drugs.

[73] Lam C.F.C., Cadelis M.M., Copp B.R. (2020). Exploration of the Electrophilic Reactiovity of the Cytotoxic Marine Alkaloid Discorhabdin C and Subsequent Discovery of a New Dimeric C-1/N-13-Linked Discorhabdin Natural Product. Mar. Drugs.

[74] Li F., Janussen D., Tasdemir D. (2020). New Discorhabdin B Dimers with Anticancer Activity from the Antarctic Deep-Sea Sponge *Latrunculia biformis*. Mar. Drugs.

[75] Li F., Pandey P., Janussen D., Chittiboyina A.G., Ferreira D., Tasdemir D. (2020). Tridiscorhabdin and Didiscorhabdin, the First Discorhabdin Oligomers Linked with a Direct C-N Bridge from the Sponge *Latrunculia biformis* Collected from the Deep Sea in Antarctica. J. Nat. Prod..

[76] Gunasekera S.P., Zuleta I.A., Longley R.E., Wright A.E., Pomponi S.A. (2003). Discorhabdins S, T, and U, New Cytotoxic Pyrroloiminoquinones from a Deep-Water Caribbean Sponge of the Genus *Batzella*. J. Nat. Prod..

[77] Samaai T., Gibbons M.J., Kelly M. (2009). A revision of the genus *Strongylodesma* Lévi (Porifera: Demospongiae: Latrunculiidae) with descriptions of four new species. J. Mar. Biol. Ass..

[78] Zou Y., Hamann M.T. (2013). Atkamine: A New Pyrroloiminoquinone Scaffold from the Cold Water Aleutian Islands *Latrunculia* Sponge. Org. Lett..

[79] Zou Y., Wang X., Sims J., Wang B., Pandey P., Welsh C.L., Stone R.P., Avery M.A., Doerksen R.J., Ferreira D. (2019). Computationally Assisted Discovery and Assignment of a Highly Strained and PANC-1 Selective Alkaloid from Alaska’s Deep Ocean. J. Am. Chem. Soc..

[80] Venables D.A., Barrows L.R., Lasotta P., Ireland C.M. (1997). Veiutamine. A New Alkaloid from the Fijian Sponge *Zyzzya fuliginosa*. Tetrahedron Lett..

[81] Samaai T., Keyzers R.A., Davies-Coleman M.T. (2004). A new species of *Strongylodesma* Levi, 1969 (Porifera; Demospongiae; Poecilosclerida; Latrunculiidae) from Aliwal Shoal on the east coast of South Africa. Zootaxa.

[82] Utkina N.K., Makarchenko A.E., Denisenko V.A. (2005). Zyzzyanones B-D, Dipyrroloquinones from the Marine Sponge *Zyzzya fuliginosa*. J. Nat. Prod..

[83] Keyzers R.A., Arendse C.E., Hendricks D.T., Samaai T., Davies-Coleman M.T. (2005). Makaluvic Acids from the South African Latrunculid Sponge *Strongylodesma aliwaliensis*. J. Nat. Prod..

[84] Genta-Jouve G., Francezon N., Puissant A., Auberger P., Vacelet J., Pérez T., Fontana A., Al Mourabit A., Thomas O.P. (2011). Structure elucidation of the new citharoxazole from the Mediterranean deep-sea sponge *Latrunculia* (*Biannulata*) *citharistae*. Magn. Reson. Chem..

[85] McCauley E.P., Smith G.C., Crews P. (2020). Unraveling Structures Containing Highly Conjugated Pyrrolo[4,3,2-de]quinoline Cores That Are Deficient in Diagnostic Proton NMR Signals. J. Nat. Prod..

[86] Stierle D.B., Faulkner D.J. (1991). Two New Pyrroloquinoline Alkaloids from the Sponge *Damiria* sp.. J. Nat. Prod..

[87] Sakemi S., Sun H.H., Jefford C.W., Bernardinelli G. (1989). Batzellines A, B and C. Novel pyrroloquinoline alkaloids from the sponge *Batzella* sp.. Tetrahedron Lett..

[88] Samaai T., Govender V., Kelly M. (2004). *Cyclacanthia* n.g. (Demospongiae: Poecilosclerida: Latrunculiidae *incertea sedis*), a new genus of marine sponges from South African waters, and description of two new species. Zootaxa.

[89] Hu J., Schetz J.A., Kelly M., Peng J., Ang K.K.H., Flotow H., Yan Leong C., Bee Ng S., Buss A.D., Wilkins S.P. (2002). New antiinfective and human 5-HT2 receptor binding natural and semisynthetic compounds from the Jamaican sponge *Smenospongia aurea*. J. Nat. Prod..

[90] Tasdemir D., Bugni T.S., Mangalindan G.C., Concepción G.P., Harper M.K., Ireland C.M. (2002). Cytotoxic Bromoindole Derivatives and Terpenes from the Philippine Marine Sponge *Smenospongia* sp.. Z. Für Nat. C.

[91] Utkina N.K., Gerasimenko A.V., Popov D.Y. (2003). Transformation of tricyclic makaluvamines from the marine sponge *Zyzzya fuliginosa* into damirones. Russ. Chem. Bull..

[92] Makarchenko A.E., Utkina N.K. (2006). UV-Stability and UV-Protective Activity of Alkaloids from the Marine Sponge *Zyzzya fuliginosa*. Chem. Nat. Comp..

[93] Aubart K.M., Heathcock C.H. (1999). A Biomimetic Approach to the Discorhabdin Alkaloids: Total Syntheses of Discorhabdins C and E and Dethiadiscorhabdin D. J. Org. Chem..

[94] Walmsley T.A., Matcher G.F., Zhang F., Hill R.T., Davies-Coleman M.T., Dorrington R.A. (2012). Diversity of bacterial communities associated with the Indian Ocean sponge *Tsitsikamma favus* that contains the bioactive pyrroloiminoquinones, Tsitsikammamine A and B. Mar. Biotechnol..

[95] Matcher G.F., Waterworth S.C., Walmsley T.A., Matsatsa T., Parker-Nance S., Davies-Coleman M.T., Dorrington R.A. (2017). Keeping it in the family: Coevolution of latrunculid sponges and their dominant bacterial symbionts. Microbiologyopen.

[96] Waterworth S.C., Parker-Nance S., Kwan J.C., Dorrington R.A. (2021). Comparative Genomics Provides Insight into the Function of Broad-Host Range Sponge Symbionts. Mbio.

[97] Jordan P.A., Moore B.S. (2016). Biosynthetic Pathway Connects Cryptic Ribosomally Synthesized Posttranslationally Modified Peptide Genes with Pyrroloquinoline Alkaloids. Cell Chem. Biol..

[98] Daniels P.N., Lee H., Splain R.A., Ting C.P., Zhu L., Zhao X., Moore B.S., van der Donk W.A. (2022). A biosynthetic pathway to aromatic amines that uses glycyl-tRNA as nitrogen donor. Nat. Chem..

[99] Wang W., Rayburn E.R., Velu S.E., Nadkarni D.H., Murugesan S., Zhang R. (2009). In Vitro and In Vivo Anticancer Activity of Novel Synthetic Makaluvamine Analogues. Clin. Cancer Res..

[100] Chen T., Xu Y., Guo H., Liu Y., Hu P., Yang X., Li X., Ge S., Velu S.E., Nadkarni D.H. (2011). Experimental Therapy of Ovarian Cancer with Synthetic Makaluvamine Analog: In Vitro and In Vivo Anticancer Activity and Molecular Mechanisms of Action. PLoS ONE.

[101] Zhang X., Xu H., Zhang X., Voruganti S., Murugesan S., Nadkarni D.H., Velu S.E., Wang M.-H., Wang W., Zhang R. (2012). Preclinical Evaluation of Anticancer Efficacy and Pharmacological Properties of FBA-TPQ, a Novel Synthetic Makaluvamine Analog. Mar. Drugs.

[102] Ireland C.M., Radisky D.C., Barrows L.R., Kramer R. (1995). Antineoplastic pyrrolo[4,3,2-de]quinoline-8(1H)-ones. U.S. Patent.

[103] Amusengeri A., Bishop Ö.T. (2019). Discorhabdin N, a South African Natural Compound, for Hsp72 and Hsc70 Allosteric Modulation: Combined Study of Molecular Modeling and Dynamic Residue Network Analysis. Molecules.

[104] Wang F., Ezell S.J., Zhang Y., Wang W., Rayburn E.R., Nadkarni D.H., Murugesan S., Velu S.E., Zhang R. (2010). FBA-TPQ, a novel marine-derived compound as experimental therapy for prostate cancer. Investig. New Drugs.

[105] Ezell S.J., Li H., Xu H., Zhang X., Gurpinar E., Zhang X., Rayburn E.R., Sommers C.I., Yang X., Velu S.E. (2010). Preclinical Pharmacology of BA-TPQ, a Novel Synthetic Iminoquinone Anticancer Agent. Mar. Drugs.

[106] Wang W., Nijampatnam B., Velu S.E., Zhang R. (2016). Discovery and development of synthetic tricyclic pyrroloquinone (TPQ) alkaloid analogs for human cancer therapy. Front. Chem. Sci. Eng..

[107] Aburjania Z., Whitt J.D., Jang S., Nadkarni D.H., Chen H., Rose J.B., Velu S.E., Jaskula-Sztul R. (2020). Synthetic Makaluvamine Analogs Decrease c-Kit Expression and Are Cytotoxic to Neuroendocrine Tumor Cells. Molecules.

[108] Levy T., Marchand L., Stroobant V., Pilotte L., Van den Eynde B., Rodriguez F., Delfourne E. (2021). IDO1 and TDO inhibitory evaluation of analogues of the marine pyrroloiminoquinone alkaloids: Wakayin and Tsitsikammamines. Bioorg. Med. Chem. Lett..

[109] Cowan J., Shadab M., Nadkarni D.H., Kailash K.C., Velu S.E., Yusuf N. (2019). A Novel Marine Natural Product Derived Pyrroloiminoquinone with Potent Activity against Skin Cancer Cells. Mar. Drugs.

[110] Dolušic E., Larrieu P., Meinguet C., Colette D., Rives A., Blanc S., Ferain T., Pilotte L., Stroobant V., Wouters J. (2013). Indoleamine 2,3,-dioxygenase inhibitory activity of derivatives of marine alkaloid tsitsikammamine A. Bioorg. Med. Chem. Lett..

[111] Hoang H., Huang X., Skibo E.B. (2008). Synthesis and in vitro evaluation of imidazole-based wakayin analogues. Org. Biomol. Chem..

[112] Lam C.F.C., Giddens A.C., Chand N., Webb V.L., Copp B.R. (2012). Semi-synthesis of bioactive analogues of the cytotoxic marine alkaloid discorhabdin C. Tetrahedron.

[113] Grkovic T., Kaur B., Webb V.L., Copp B.R. (2006). Semi-synthetic preparation of the rare, cytotoxic, deep-sea sourced sponge metabolites discorhabdins P and U. Bioorg. Med. Chem. Lett..

[114] Nijampatnam B., Nadkarni D.H., Wu H., Velu S.E. (2014). Antibacterial and Antibiofilm Activities of Makaluvamine Analogs. Microorganisms.

[115] Furrow F.B., Amsler C.D., McClintock J.B., Baker B.J. (2003). Surface sequestration of chemical feeding deterrents in the Antarctic sponge *Latrunculia apicalis* as an optimal defense against sea star spongivory. Mar. Biol..

